# Evolution to a Competency-Based Training Curriculum for Pharmaceutical Medicine Physicians in Switzerland

**DOI:** 10.3389/fphar.2019.00164

**Published:** 2019-02-27

**Authors:** Gabriel Schnetzler, Markus F. Bremgartner, Regina Grossmann Straessle, David Spirk, Fabian Tay, Rahel Troxler Saxer, Martin Traber

**Affiliations:** ^1^F. Hoffmann-La Roche Ltd., Basel, Switzerland; ^2^Bexa Medical Services, Kirchberg, Switzerland; ^3^Mundipharma Medical Company, Basel, Switzerland; ^4^Clinical Trials Center, University Hospital of Zürich, Zurich, Switzerland; ^5^Institute of Pharmacology, University of Bern, Bern, Switzerland; ^6^Sanofi S.A., Geneva, Switzerland; ^7^Pfizer, Zurich, Switzerland

**Keywords:** Pharmaceutical Medicine, competency-based, post-graduate training, board-certification, vocational training, Switzerland

## Abstract

In Switzerland, Pharmaceutical Medicine has existed as one of 46 physician specialties accredited by the Federal Office of Public Health for more than 20 years. As a medical-scientific discipline, our goal is to enable best possible therapeutic coverage for the benefit of patients and society through a medical need-based development and optimal use of medicinal products. The role of the specialist in Pharmaceutical Medicine is to closely collaborate with various stakeholders of the healthcare system in the context of the discovery, research, development and approval of new medicinal products, as well as safe and effective use of new and established medicinal products in daily clinical practice. The post-graduate training consists of 2 years of patient-related clinical work, followed by 3 years of vocational training at certified training centers in Pharmaceutical Medicine. This also includes completion of an academic post-graduate diploma in Pharmaceutical Medicine (30 ECTS) according to the IFAPP/PharmaTrain syllabus and a 1 day board exam. As part of an ongoing revision of the training curriculum, we are developing a Swiss Catalog of Core Competencies in Pharmaceutical Medicine (SC^3^-PM), based on the IFAPP competency framework for drug development specialists in industry. In this article we discuss how we adapt the scope of the IFAPP competency framework to better reflect such roles in academic institutions or regulatory bodies in Switzerland.

## Pharmaceutical Medicine Training in Switzerland

On January 1, 1999, the Swiss Department of Health officially recognized Pharmaceutical Medicine as a fully board-certified physician specialty in Switzerland. This marked a key milestone for the Swiss Society of Pharmaceutical Medicine (SSPM). The SSPM was founded mid 1997 with the aim of advancing the science and practice of Pharmaceutical Medicine, by developing and maintaining competencies, ethics and integrity in order to provide the highest professional standards for the benefit of the patients and public (Traber and Althaus, 2010). Today, Pharmaceutical Medicine is one of 46 physician specialties accredited by the Swiss Department of Health (Swiss Institute for Post-Graduate Training and Continuous Education, 2018c), and Switzerland is still one of the few countries where such a board-certified physician specialty exists (Nell, 2018).

A board-certified specialist title is the reference for the post-graduate qualification of a physician in Switzerland and is publicly disclosed in the national registry of medical professionals (Federal Office of Public Health, 2018b). For most specialties it is the pre-requisite to practicing independently and being remunerated for related clinical activities. There are no clinical or regulatory activities restricted to a physician who is specialized in Pharmaceutical Medicine, however, the title serves as evidence of the qualifications required by the Swiss Clinical Trials Ordinance to act as sponsor-investigator (Swiss Clinical Trials Ordinance, 2017).

Since its introduction, the training curriculum for Pharmaceutical Medicine requires 2 years of patient care related clinical training, followed by 3 years of vocational training at certified training centers for Pharmaceutical Medicine (reviewed in Traber and Althaus, 2010). This general educational framework for postgraduate training for physicians in Switzerland is governed by the Swiss Institute for Post-Graduate Training and Continuous Medical Education (Schweizerisches Institut für Weiter- und Fortbildung, SIWF), while the SSPM is responsible for the subject specific content of the curriculum (see Supplementary Figure S1) (Swiss Institute for Post-Graduate Training and Continuous Education, 2018b). Training centers are classified in four categories (A–D) based on multiple parameters such as the number of board-certified (or comparably qualified) educational staff as well as metrics and infrastructure regarding various domains offered for training from the broad spectrum of Pharmaceutical Medicine.

Historically, clinical trials were defined as the core element of medical product development, which has been reflected in the educational goals and requirements. Trainees had to demonstrate at least 2 years of project level involvement in clinical trials and training centers were classified based on number of CRFs actively managed in phase I–IV trials, as well as metrics on adverse event reports. Training center activity in the area of drug discovery, pre-clinical development, pharmacological development and public health had a lower weight. The main focus on clinical trials has gradually been removed and since the last revision in 2016 all core areas are contributing now in a balanced way to the vocational training for Pharmaceutical Medicine (Swiss Institute for Post-Graduate Training and Continuous Education, 2018d) (see Supplementary Table S2).

Each training site has to prepare its own concept for vocational training based on the educational goals outlined in the current training curriculum for Pharmaceutical Medicine (Swiss Institute for Post-Graduate Training and Continuous Education, 2018d). This serves as the basis for validation through a SIWF-led expert panel (including one SSPM representative), which grants approval of new training sites, renewal in case of change of training center lead at an approved site, or general periodic audits (at least every 7 years). Additionally, each trainee is required to attend and document a minimum of 360 h of theoretical training on the topics of discovery (12 h), pharmaceutical development (16 h), pre-clinical development (24 h), clinical development (150 h), pharmacovigilance (32 h), medical information (32 h), drug regulations (46 h), socioeconomics and public health (24 h) as well as management (24 h). This can be substituted by successful completion of an academic post-graduate diploma course following the IMI/PharmaTrain syllabus (PharmaTrain, 2018), which is offered in Switzerland by the European Center for Pharmaceutical Medicine (ECPM) (European Center for Pharmaceutical Medicine, 2018) at the University of Basel. Learning progress is evaluated by the training center leads using regular structured feedback and workplace related assessments (at least four times per year) and documented in a central e-logbook during the entire training period.

The curriculum allows a maximum of 1 year of training at an accredited training center for Prevention Medicine and Public Health, or in Clinical Pharmacology and Toxicology. Equally, 1 year research as part of an MD/PhD program, or under the supervision of a certified study sponsor-investigator outside of a training center for Pharmaceutical Medicine is accepted. No more than 1.5 years of training outside of Switzerland can be accounted for the curriculum. However, mutual recognition of foreign specialty titles in Pharmaceutical Medicine exists for countries with a similar training profile (e.g., Faculty of Pharmaceutical Medicine, 2018).

Currently, board examination consists of an extensive knowledge test (120 questions, multiple choice) followed by an assessment based on a written essay on three open formulated questions (2 h) and an interview based discussion (approx. 1 h) covering a published clinical research paper as well as other topics of Pharmaceutical Medicine. Under exceptional circumstances, a specialist title can be granted based on merits due to pioneering work in the field of Pharmaceutical Medicine.

A board-certified specialist has an obligation to complete 80 h of continuous medical education (CME) per year. Of these at least 50 h need to be testified through CME credits, of which half need to be in the field of Pharmaceutical Medicine. Importantly, CME credits in any other specialty can be recognized, if it is linked to the therapeutic field of daily work (e.g., CME in cardiology if involved in medicinal product development in cardiovascular diseases). CME credits must be self-recorded in an online system operated by the SIWF and completion of above mentioned requirements need to be certified periodically in 3 year intervals (Swiss Institute for Post-Graduate Training and Continuous Education, 2018a).

## Trends in Pharmaceutical Medicine Training in Switzerland During the Past 20 years

Over the past 20 years, more than 120 physicians have been board-certified in Pharmaceutical Medicine (Supplementary Figure S3). The average number of physicians completing their training fluctuated around three to five per year. Only at time of introduction of this specialty title (1999–2003), a higher number of physicians working already in the field of pharmaceutical medicine were certified based on their “merits” or through mutual recognition programs.

Initially, most specialists have been trained in industry, however, over the past 10 years there has been a surge in specialists trained at academic centers (Supplementary Figure S4). This coincides with the emergence of clinical trial units (CTUs) at university hospitals and other tertiary care centers, followed by the establishment of the Swiss Clinical Trial Organization (SCTO) in 2009 with the concomitant improvement of clinical trial quality and metrics (Von Niederhäusern et al., 2018). The number of certified training centers for Pharmaceutical Medicine based in the academic setting in Switzerland increased during this period from one to three.

At the same time, the number of certified training centers among the local affiliates of pharmaceutical companies declined in Switzerland. This can be attributed to two key reasons: First, a decline of physicians who select industry (and especially local medical affairs departments of pharmaceutical company affiliates in Switzerland) as a workplace for their post-graduate training. The absence of a trainee for more than 3 years leads to suspension of the training center certification. Second, several training centers lost their accreditation despite the presence of potential training candidates, because the head of the training center did not have the required qualifications. Additionally, the trend for internationalization and fragmentation of the different functions (research and development, medical affairs, regulatory affairs, pharmacovigilance, patient access, medical information), where reporting and governance structures are no longer maintained at local level, increases complexity for country medical directors to prepare and maintain a vocational training concept according to the curriculum.

Several pharmaceutical companies have their international head quarters or large operating centers including research and development, or medical affairs organizations based in Switzerland. Despite substantial investment in training and mentoring of employees, none of these are certified sites for vocational training. Administrative hurdles and constraints imposed by the Swiss post-graduate training process for physicians together with a lack of corporate interest and perceived benefit have anecdotally been quoted as reasons not to pursue.

## Evolution to a Competency-Based Training Curriculum for Pharmaceutical Medicine in Switzerland

The training curriculum itself is also subject to a regular accreditation process according to the Swiss Medical Professions Act. This was most recently conducted between February 2016 and August 2018. It involved a critical self-reflection on the purpose and content of the post-graduate training curriculum through the SSPM, as well as an external review by independent international experts. During this process we identified the need to further evolve the original knowledge based curriculum with “learning based” to a competency- based approach, aligned with the updated IFAPP syllabus and competency statement (IFAPP, 2016). Accreditation was granted and an updated curriculum has to be in place by end of 2019, which addresses the following additional requirements (Federal Office of Public Health, 2018a):

(a) A vision and mission statement has to be defined, which outlines the role of a specialist in Pharmaceutical Medicine and his or her interactions with other stakeholders of the healthcare system regarding to contribution of care.
(b) A plan or method to maintain the high quality of training in order to react and adapt to the evolving environment in healthcare.

The SSPM has established a new working group involving the heads of selected training centers to update the training curriculum through a series of workshops and off-line review cycles. Here we outline the core aspects of the revised curriculum and share the proposed changes.

### The Role of the Specialist in Pharmaceutical Medicine in the Swiss Healthcare System

As a medical-scientific discipline, our vision is to provide the best possible therapeutic coverage for the benefit of patients and society through a medical need-based development and optimal use of medicinal products. The role of the specialist in Pharmaceutical Medicine is to closely collaborate with various stakeholders of the healthcare system in the context of the discovery, research, development and approval of new medicinal products, as well as safe and effective use of new and established medicinal products in daily clinical practice. The patient-centric, evidence-based decision process has a direct influence on the therapeutic coverage of patients, potentially beyond our national healthcare system.

A patient-centric development of medicinal products has to adhere to stringent legal, ethical and qualitative requirements with regards to the planning and conduct of clinical trials, accurate interpretation of pre-clinical, toxicological, pharmacological and clinical results with appropriate consideration of the benefit-risk profile and socio-economic aspects. The knowledge and competence in preparing documents for regulatory (e.g., clinical trial applications, marketing authorization application, periodic safety update reports) or reimbursement (e.g., health technology assessments) submissions, the continuous improvement of therapeutic benefit through appropriate medical information, supply coverage, appropriate risk management through the whole lifecycle of medicinal products requires expertise in Pharmaceutical Medicine. Therefore, the work of specialists in Pharmaceutical Medicine is valuable:

- In translational medicine and clinical research at academic centers and hospitals (incl. dedicated CTUs and general clinical services).
- At manufacturers and distributors of pharmaceutical or biomedical products and their service providers (including contract research organizations and consultancies).
- With regulatory and related competent authorities (e.g., Swissmedic, Federal Office of Public Health).

### Revision to the Curriculum Structure

**FIGURE 1:**
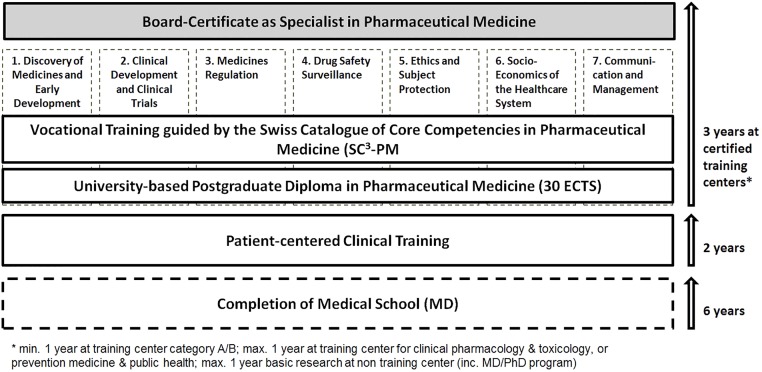
Illustration of the training curriculum and requirements to become board-certified specialist in Pharmaceutical Medicine in Switzerland.

While the general structure of post-graduate training will remain the same (Figure 1), we propose a few procedural changes to strengthen the medical-scientific character of our discipline. A university-based post-graduate course with the completion of the Basis Diploma in Pharmaceutical Medicine according to the IMI/PharmaTrain syllabus (min 30 ECTS) (PharmaTrain, 2018) shall become the standard. We believe that peer-to-peer learning and exchange with colleagues from other areas of Pharmaceutical Medicine provide additional value over purely internal courses at training sites. In addition it aligns with the structure of the PharmaTrain Specialist in Medicines Development Certification Program (PharmaTrain, 2014).

The requirement for publication of an individual scientific or clinical research work in a peer-reviewed journal for the board-certification is currently debated. While the competency of medical writing (including assessments for medical information and preparation of regulatory documents) is being well trained at the centers, there is often limited opportunity to conduct original research. To improve scientific exchange we intend to mandate the participation in at least one meeting co-organized by the SSPM during the time of the specialist training. These meetings include the Annual Symposium in Pharmaceutical Medicine co-sponsored with the Swiss Association of Pharmaceutical Professionals (SwAPP), a 1 day event with state-of-the-art lectures and discussions on current topics, or the annual Spring Meeting organized by the Swiss Society of Pharmacology and Toxicology (SSPT). The SSPT is the umbrella organization of several scientific societies in this field. The aim of this 1 day event is sharing original scientific work, where active contribution is additionally incentivized through awards.

Finally, we would like to pursue the idea of an “exchange” program, where all trainees meet regularly for joint sessions and rotate among the affiliated training centers. During these half-day workshops, the trainee(s) elaborate one or more specific case studies under the supervision of the training head of the hosting center. The aim of such sessions would be to share practice and working experience between the different types of centers (e.g., academia- industry).

### Defining the Swiss Catalog of Core Competencies in Pharmaceutical Medicine (SC^3^-PM)

During the recent accreditation process we have proposed to evolve the current knowledge based curriculum to a competency based curriculum. Therefore we are preparing a Swiss Catalog of Core Competencies in Pharmaceutical Medicine (SC^3^-PM) which adopts the concept of the IFAPP core competency description based on applied knowledge, skills and behaviors: We are currently adapting the proposed content in the domains of (1) Discovery of Medicines and Early Development, (2) Clinical Development and Clinical Trials, (3) Medicines Regulation, (4) Drug Safety Surveillance, (5) Ethics and Subject Protection, (6) Socio-Economics of the Healthcare System, and (7) Communication and Management (Table 1). Although refinement is still in progress, we would like to share already some of the thoughts and feedback received so far as part of the initial consultation:

(a) The current IFAPP statements focus on specialists (including non-physicians) working mainly on drug development in industry. However, we are convinced that the competencies have to equally apply to physician specialists working in academic institutes or regulatory bodies. Hence, we are removing or replacing industry specific aspects to reflect the broader scope. Particularly competencies on commercial aspects shall be substituted by a more socio-economic view on the healthcare system, while business specific expertise can be acquired through other post-graduate programs (e.g., Master of Business Administration).
(b) We propose some revisions to better reflect the current issues and topics beyond development of medicinal products. Especially aspects on the competencies of a specialist in Pharmaceutical Medicine to enable access to medicines have been suggested. This includes expected knowledge, skills and behaviors with respect to health technology assessments and reimbursement. It also refers to situations, where supply of medicines could be limited or interrupted for various reasons. Further, we consider strengthening the aspect of medical information and appropriate communication with stakeholders along the whole lifecycle of a medicinal product.
(c) Ensuring compliance with Swiss law and regulations where appropriate.

**Table 1:** Proposed competency statements for a specialist in pharmaceutical medicine (SPM) in Switzerland.

Domain	Core competency statements
(1) Discovery of medicines and early development	The SPM is able to identify unmet therapeutic needs, evaluate the evidence for a new candidate for clinical development and design a Clinical Development Plan (CDP) for a Target Product Profile (TPP).
(2) Clinical development and clinical trials	The SPM is able to design, execute and evaluate exploratory and confirmatory clinical trials and prepare manuscripts or reports for publication and regulatory submissions.
(3) Medicines regulation	The SPM is able to interpret effectively the regulatory requirements for the clinical development of a new drug through the product life-cycle to ensure its appropriate therapeutic use and proper risk management
(4) Drug safety surveillance	The SPM is able to evaluate the choice, application and analysis of post-authorization surveillance methods to meet the requirements of national/international agencies for proper information and risk minimization to patients and clinical trial subjects.
(5) Ethics and subject protection	The SPM is able to combine the principles of clinical research and business ethics for the conduct of clinical trials and commercial operations within the organization.
(6) Socio-economics of the healthcare system	The SPM is able to appraise the reasonable development and use of diagnostic, prophylactic and therapeutic means for the care of healthy volunteers and patients, thereby promoting the efficient use of available resources within the legal boundaries.
(7) Communication and management	The SPM is able to use the required skills for effective communication and management across stakeholders of the healthcare system, including clinical setting (e.g., patients, care givers, prescribers), competent authorities and interdisciplinary teams at the workplace.


## Challenges, Plan of Action, Learnings and Outlook

A defined post-graduate training path and qualification process for physicians in Switzerland to obtain a recognized specialty title in Pharmaceutical Medicine has become well established in the course of the past 20 years. This has also recently been validated by the FOPH’s re-accreditation process (Federal Office of Public Health, 2018a). However, this requires adherence to the general framework and certain operational aspects of post-graduate training for all physicians in Switzerland. While the concept for competency-based training was introduced by the SIWF several years ago within the overarching ordinance for physician post-graduate training, the IFAPP model now helps us to better define targets for applied knowledge, skills and behaviors regarding Pharmaceutical Medicine. Moving forward, we believe that the currently defined tools [Mini-Clinical Evaluation Exercise (Mini-CEX) and Direct Observation of Procedural Skills (DOPS)] can be adapted and adequately used for the required quarterly evaluation of the training progress and acquired competencies (Swiss Institute for Post-Graduate Training and Continuous Education, 2019b). At this stage we see limited opportunities to deviate from the defined structures for the final board examinations, other than possible adaptations in the questions to further explore competencies in the dimensions of “skills” and “behaviors.” We will also continue to implement the structure and methodology provided by the SIWF for evaluation and documentation of competencies as part of the continuous professional development (Swiss Institute for Post-Graduate Training and Continuous Education, 2019a).

Drug development and lifecycle management of both, new and established medicinal products is a multidisciplinary endeavor involving professionals with different educational background in life sciences. The Swiss Association for Pharmaceutical Professionals (SwAPP) provides to non-physicians a similar 5-year certification program in Pharmaceutical Medicine following the IFAPP/Pharmatrain syllabus (Swiss Association of Pharmaceutical Professionals, 2019). Despite comparable expertise, certain clinical trial related activities (e.g., clinical investigator) are restricted by law to trained physicians.

As a result of the accreditation process described initially, the SSPM has formed a working group with the heads of selected training centers to ensure regular exchange and dialog with the aim to improve objectives and quality of the vocational training across the different sites. We also intend to leverage areas of special interest and expertise of the various centers to allow for better exchange and peer-to-peer learning through rotation programs for trainees. Furthermore, this working group will serve as a platform to address evolving training needs and adaptation of the curriculum, namely for emerging trends such as personalized healthcare, big data and artificial intelligence, as well as to address new therapeutic approaches using gene-editing technologies. Most importantly, we are on track for timely implementation of this a new competency-based training curriculum for Pharmaceutical Medicine in Switzerland to build up the next generation of specialists.

## Author Contributions

GS and MT prepared a first draft. All authors actively contributed in review and finalization of the manuscript.

## Conflict of Interest Statement

All authors are members of the SSPM and involved in the revision of the training curriculum for Pharmaceutical Medicine in Switzerland. No funding has been received for this work. All authors are employed by the institutes or companies of their affiliation; however, this initiative is independent of employer or affiliation.
