# Supplementary material for: Evolution to a Competency-Based Training Curriculum for Pharmaceutical Medicine Physicians in Switzerland
Source: Front Pharmacol. 2019 Feb 27;10:164. doi: 10.3389/fphar.2019.00164 (PMC6400888; doi:10.3389/fphar.2019.00164)
Supplement: Supplementary file 1 [file Data_Sheet_1.pdf]

Supplementary Materials

Figure S1: Simplified structure of governance for post-graduate (PG) training and continuous medical education (CME) for physicians specializing in Pharmaceutical Medicine in Switzerland.

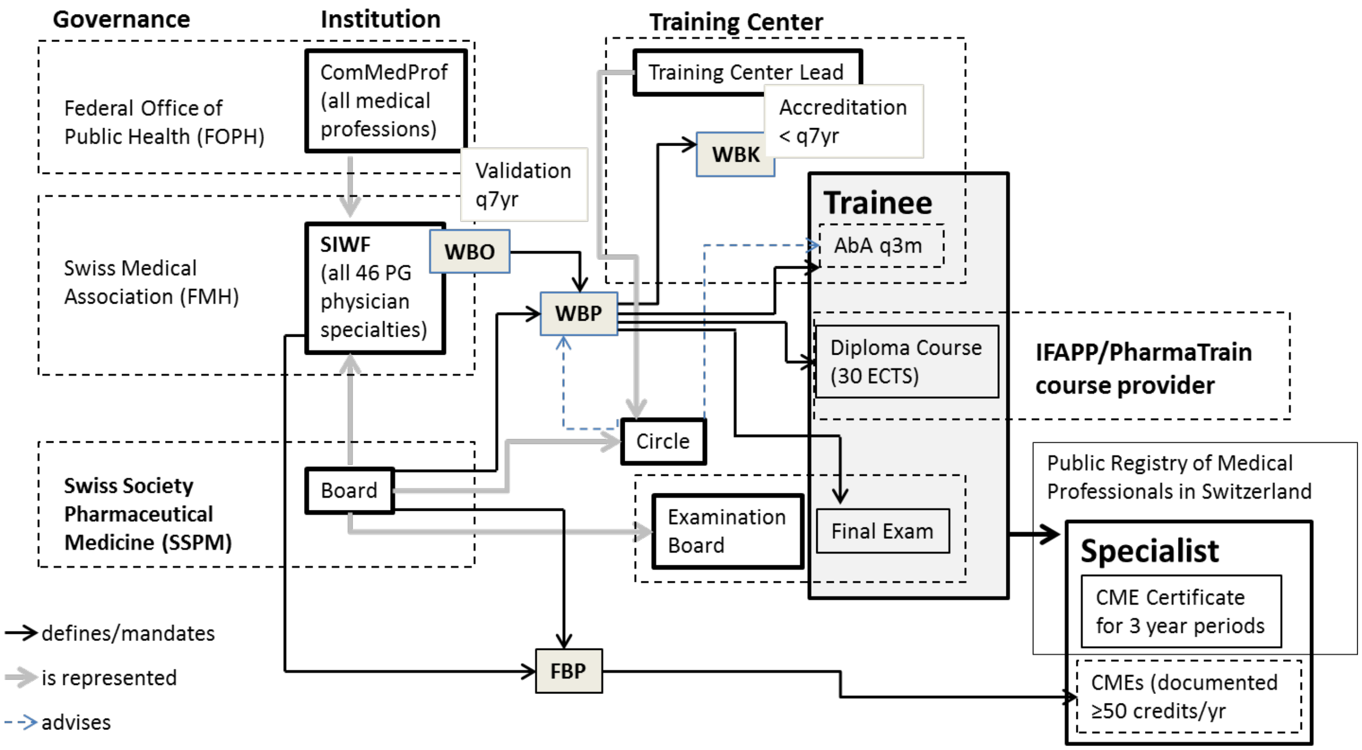

Swiss law specifically regulates certain medical professions: Physicians, Dentists, Pharmacists, Osteopaths and Veterinarians. Delegated by the FOPH, the Commission on Medical Professions (ComMedProf) oversees graduate, post-graduate and continuous medical education. For physicians, the Swiss Medical Association (FMH) created the Swiss Institute for Postgraduate and Continuous Profession Education (Schweizerisches Institut für Weiter- und Fortbildung, SIWF), which governs the PG and CME for the 46 accredited physician specialties. The SIWF, therefore, represents all relevant stakeholders, including at least one delegate of each physician specialty society. The SIWF defines the overarching Ordinance for PG training (Weiterbildungsordnung, WBO), which includes the general framework for the PG training and CME of all physician specialties and defines the general competencies as a "medical expert" using the CanMEDS model. The SIWF provides the required tools to assess training progress for the workplace-based assessments (Arbeitsplatz basierte Assessments, AbA) and a secure central digital platform (e-log book for trainees and later for CME credit declaration).

The Swiss Society of Pharmaceutical Medicine (SSPM) represents the respective specialty in the SIWF and their commissions and is responsible for the related content of the PG training program (Weiterbildungsprogramm, WBP) and continuous professional development (Fortbildungsprogramm,

FBP). The FOPH runs every 7 years an accreditation process for the PG training program of each medical specialty (latest process completed August 2018). The SIWF (including a subject matter expert from the SSPM) validates each PG training center and their PG training concept (Weiterbildungskonzept, WBK) in case of an application of a new site, change of the training center lead, or in a periodical manner (at least every 7 years).

Diplomas in Pharmaceutical Medicine from IFAPP/PharmaTrain accredited providers (30 ECTS) are recognized as theoretical training mandated by the WBP.

The SSPM runs the Board exams, and upon completion of the all PG training requirements, the SIWF (upon recommendation by the SSPM) awards the specialty title and later also the CME certifications (for 3 year periods). The documentation and validation of at least 50 credits per year is required by the CME guidance (Fortbildungsprogramm, FBP), resulting in a certification status which is disclosed in the national registry of medical professionals in Switzerland (www.medregom.admin.ch).

A recently formed working group composed of the heads of selected training centers (“Circle”) supports the SSPM with regards to the PG training curriculum content (Swiss Catalogue of Core Competencies in Pharmaceutical Medicine (SC3-PM)) and methods for regular assessment of training progress.

Table S2: Evolution on specifications to categorize vocational training centers.

Implementation (year)	1999				2009				2016			
**Category**	A	B	C	D	A	B	C	D	A	B	C	D
Max. yrs accountable°	3	2	1	½	3	2	1	½	3	2	1	½
Number of Mentors* (n)	2	1	1	1	2	1	1	1	2	1	1	1
Number of Trainees (≥n)	1	1	1	1	1	1	1	1	1	1	1	1
Clinical studies phI-III	100[a]	60	60	-	x	x						
Clinical studies phIV	250[a]	150	150	-	x	x						
Pharmacovigilance	50[b]	30	30	-	x	x						
Registration	100[c]	60	60	-	plus ≥2 core areas covered	plus 1 core area covered	3 core areas covered	1 core area covered	≥5 core areas covered	4 core areas covered	3 core areas covered	1 core area covered
Medical Information	100[c]	100	60	-								
Discovery				x								
Pharmacologic Development				x								
Preclinical Development				x								
Socio-economics/Public Health				x								
Number of core areas (n)	5	4	3	1	5	4	3	1	5	4	3	1
Journal Club (n/month)									1	1		
Structured Teaching (hrs/week)"									3	2	1	

*Physician Board-certified in Pharmaceutical Medicine; or equivalent expertise (exceptionally)
°For total of 3 years vocational training
"incl. time spent on courses (e.g. Diploma Course in Pharmaceutical at European Center of Pharmaceutical Medicine University Basel, ECPM)
a) case report forms; b) adverse event reports; c) number of registered medicinal products

Figure S3: Current working area of Swiss board-certified specialists in Pharmaceutical Medicine (Source: Database of the Swiss Institute for Post-Graduate Training and Continuous Medical Education, as of Nov 28, 2018)

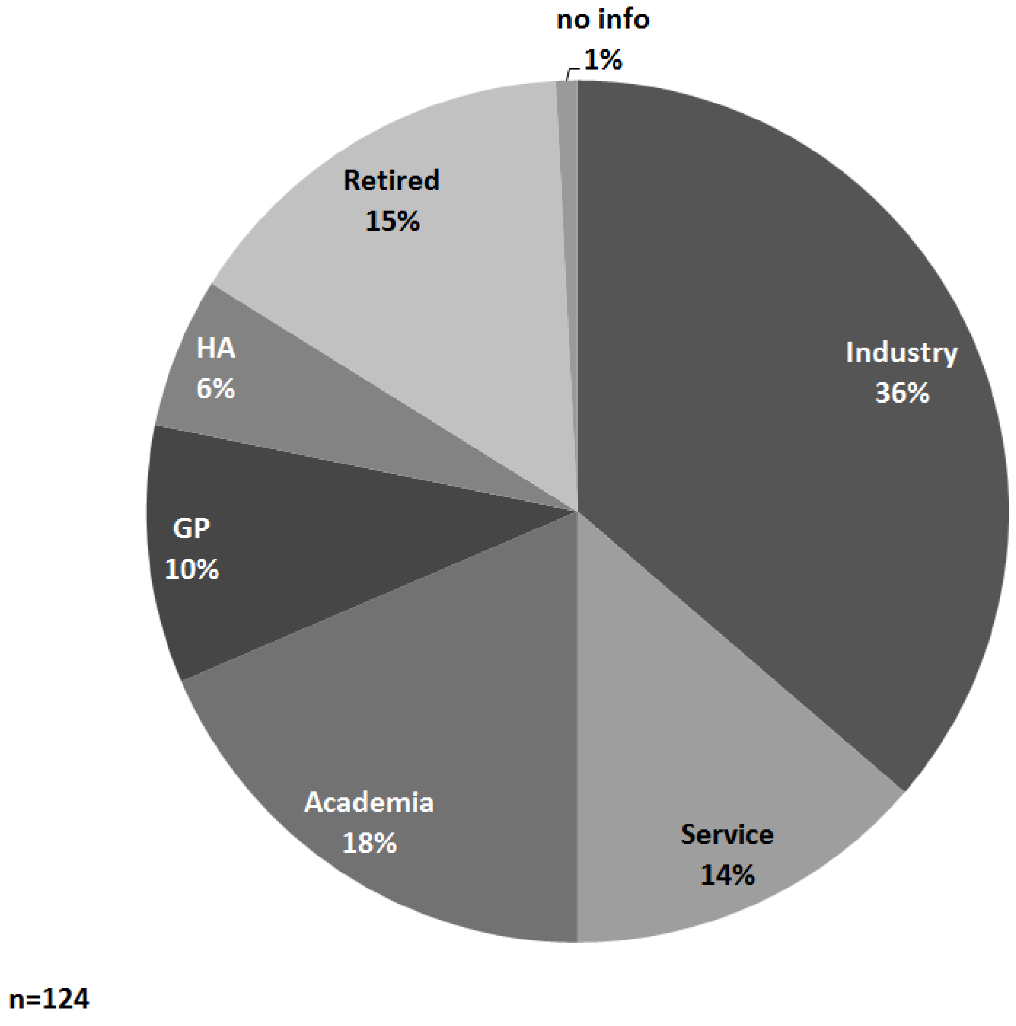

GP, general practitioner; HA, Health Authorities; Service, service providers incl. consultancies and contract research organizations

Figure S4: Distribution of trainings- or workplace of candidates at time of board certification. (Source: Database of the Swiss Institute for Post-Graduate Training and Continuous Medical Education, as of Nov 28, 2018)

In the earlier years of this specialty existence, a substantial number of titles were awarded based on merits (to existing experts and pioneers in Pharmaceutical Medicine) rather than through a defined training curriculum.

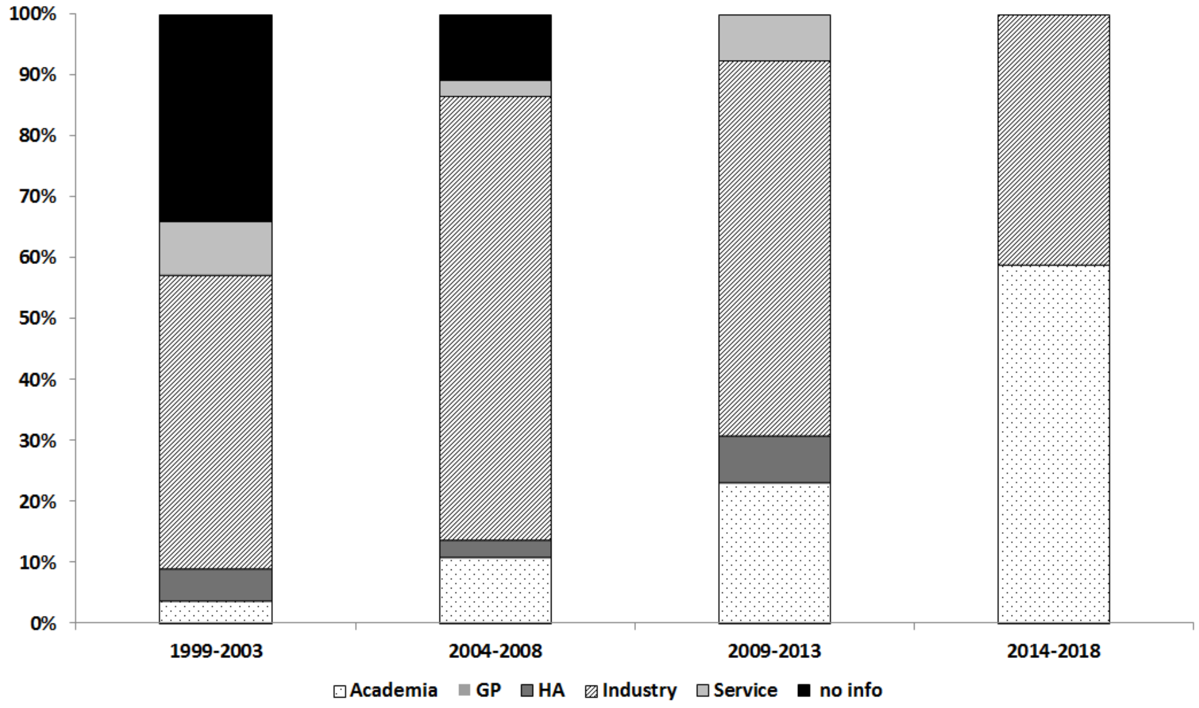

GP, general practitioner; HA, Health Authorities; Service, service providers incl. consultancies and contract research organizations
